# Fluorescence in situ hybridisation in Carnoy’s fixed tonsil tissue

**DOI:** 10.1038/s41598-022-16309-w

**Published:** 2022-07-20

**Authors:** S. T. Clark, S. Waldvogel-Thurlow, B. Wagner Mackenzie, R. G. Douglas, K. Biswas

**Affiliations:** Department of Surgery, Faculty of Medical and Health Sciences, The University of Auckland, Grafton, Auckland 1023 New Zealand

**Keywords:** Immunology, Microbiology, Molecular biology, Medical research, Pathogenesis

## Abstract

Fluorescence in situ hybridisation (FISH) is a powerful molecular technique that enables direct visualisation of specific bacterial species. Few studies have established FISH protocols for tonsil tissue in Carnoy’s fixative, accordingly limiting its application to investigate the pathogenesis of tonsillar hyperplasia. Tonsil tissue from 24 children undergoing tonsillectomy for either recurrent tonsillitis or sleep-disordered breathing were obtained during a previous study. The specificity of each of the five FISH probes (*Fusobacterium* spp*.*, *Bacteroides* spp., *Streptococcus* spp., *Haemophilus influenzae* and *Pseudomonas* spp.) were successfully optimised using pure and mixed bacterial isolates, and in Carnoy’s fixed tonsil tissue. *Bacteroides* spp. were present in 100% of patients with microcolonies. In comparison, the prevalence of *Fusobacterium* spp. was 93.8%, *Streptococcus* spp. 85.7%, *H. influenzae* 82.35% and *Pseudomonas* spp. 76.5%. Notable differences in the organisation of bacterial taxa within a single microcolony were also observed. This is the first study to establish a robust FISH protocol identifying multiple aerobic and anaerobic bacteria in Carnoy’s fixed tonsil tissue. This protocol provides a strong foundation for combining histological and microbiological analyses of Carnoy’s fixed tonsil samples. It may also have important implications on the analysis of microorganisms in other human tissues prepared using the same techniques.

## Introduction

Over recent decades, new culture-independent techniques have been developed to investigate the human microbiome. Fluorescence in situ hybridisation (FISH) is one molecular technique that applies oligonucleotide probes to target genes unique to bacterial species of interest^1^. The main advantages of FISH are its ability to accurately identify bacteria and provide a two dimensional visualisation of the microorganisms in situ. Furthermore, FISH techniques can be combined with confocal laser scanning microscopy to visualise individual cells of bacterial species within dense and diverse bacterial communities^1^.

Despite these known advantages, very few studies have applied FISH to tonsil tissue. In 2007, Swidsinki et al. were the first to publish a paper investigating the spatial organisation of bacteria in quiescent adenoiditis and tonsillitis using FISH^2^. This study applied an extensive set of 51 different FISH probes to Carnoy’s fixed tonsil tissue, however, the paper was not accompanied by a published methods protocol and accordingly not repeated^2^. Furthermore, Swidsinski et al. investigated the general features of a tonsil, and did not focus on the specific characteristics and composition of bacterial microcolonies^3^. Large numbers of B lymphocytes and T lymphocytes have been previously observed around bacterial microcolonies in the tonsillar crypts of patients with tonsillar hyperplasia (TH)^3^. These findings suggest a potential role of microcolonies in the chronic inflammation and recurrent infection that is characteristic of TH. Shortly thereafter, Heiniger et al., developed a method to detect *Moraxella catarrhalis* in adenoids and tonsils using FISH, however, this protocol was limited to paraformaldehyde (PFA) fixed tonsil tissue^4^. In 2009, Nistico et al. published the first formal FISH protocol to aid the detection of biofilm in the middle ear and upper respiratory tract mucosa^1^. Two additional papers were later published, using adaptations of the same protocol^5, 6^. However, all three papers were again limited to PFA or 5% glutaraldehyde/0.1 M Sorensen’s phosphate buffer (pH 7.4) fixed tonsil tissue. Two final papers utilising FISH in tonsil tissue were published by Zautner et al.^7^ and Stepinska et al.^8^. Again, both were limited to PFA fixed tonsil tissue^7, 8^. Furthermore, Zautner et al. only utilised a *Staphylococcus aureus* FISH probe^7^ and Stepinska et al. tailored their protocol for use on suspended tonsil tissue in order to better identify intracellular bacteria^8^.

To our knowledge, FISH techniques have not yet been applied to detect a range of bacterial species within dense tonsillar microcolonies in Carnoy′s fixed tonsil tissue. In this study a protocol was developed for tonsil specimens fixed in Carnoy’s fixative, embedded in paraffin, utilising four genus-specific FISH probes (*Bacteroides* spp. (BAC303), *Fusobacterium* spp. (FUSO), *Pseudomonas* spp. (PSE227) and *Streptococcus* spp. (STRC493)) and one species-specific FISH probe (*H. influenzae* (HAEinf)) selected based on findings in a previous study by our group^9^. Implementing this technique in tonsil tissue may provide critical insights into differences in the spatial arrangement of potentially pathogenic bacteria within microcolonies for a broad range of aerobic and anaerobic species.

## Methods

### Fluorescence in situ hybridisation (FISH) probe selection

FISH probes for five highly abundant and potentially pathogenic bacteria in tonsils were chosen for optimisation and analysis in bacterial microcolonies using probeBase ^10^. The genera specific probes included BAC303^11^, FUSO^12^, STRC493^13^, and PSE227^14^, in addition to the species-specific probe HAEinf^15^. Further information about each of these probes is outlined in Table 1. Each FISH probe was synthesised with a fluorescein isothiocyanate (FITC) or a carbocyanine dye (Cy3 or Cy5) at the 5′ end of the oligonucleotide probe (Integrated DNA Technologies PTE, Singapore). Each taxa-specific probe was stained in combination with both DAPI (4′6-Diamidino2-Phenylindole) (Dihydrochloride, Molecular Probes Eugene Oregon, USA, 1:1000 dilution) and a universal bacterial probe (EUB338^16^). This combination verified the presence of a microcolony and assisted with visualisation. All stock solutions for each probe were diluted with double-distilled water (ddH_2_O), to give a final concentration as outlined below in Table 1.

**Table 1:** Bacterial fluorescence in situ hybridisation (FISH) probes and dilutions.

Probe	Target bacteria	Genetic sequence	Concentration (µM)	Dyes
BAC303^11^	*Bacteroidaceae* spp. *Prevotellaceae* spp.*,* some *Porphyromonadaceae* spp.	5′- CCA ATG TGG GGG ACC TT -3′	10	Cy3, Cy5
FUSO^12^	*Fusobacterium* spp.	5′- CTA ATG GGA CGC AAA GCT CTC -3′	10	Cy3, Cy5
STRC493^13^	*Streptococcus* spp.*,* some *Lactococcus* spp.	5′- GTT AGC CGT CCC TTT CTG G -3′	10	Cy3, Cy5
PSE227^14^	*Pseudomonas* spp.	5′- AAT CCG ACC TAG GCT CAT C -3′	10	Cy3, Cy5
HAEinf^15^	*Haemophilus influenzae*	5′- CCG CAC TTT CAT CTT CCG -3′	10	Cy3, Cy5
EUB338^16^	most bacteria	5′- GCT GCC TCC CGT AGG AGT -3′	5	FITC

*Cy3* carbocyanine dye 3, *Cy5* carbocyanine dye 5, *FITC* fluorescein isothiocyanate.

### Bacterial cell culture

Pure bacterial cell cultures were used as positive controls to evaluate the specificity of each FISH probe, as outlined in Table 2. *B. fragilis*, *F. nucleatum* and *H. influenzae* were selected from the medical section of the New Zealand reference culture collection at the Institute of Environmental Science and Research Limited (ESR, New Zealand). *S. aureus* was isolated from a human sinonasal cavity at the LabPLUS medical laboratory at Auckland City Hospital as part of a previous study^17^. Standard laboratory strains of *P. aeruginosa*, *S. pyogenes* and *E. coli* were obtained from the Department of Molecular Medicine & Pathology, University of Auckland.

**Table 2:** Bacterial cell cultures used in this study to test and optimise FISH probes.

Bacterial species	Strains	Source
*Bacteroides fragilis*	ATCC 25,285	ESR
*Escherichia coli*	K-12 DH5alpha, ATCC PTA-4079	LabPLUS
*Fusobacterium nucleatum*	ATCC 25,586	ESR
*Haemophilus influenzae*	ATCC 10,211 type b	ESR
*Pseudomonas aeruginosa*	ATCC BAA-47	UoA
*Streptococcus pyogenes*	ATCC 700,294	UoA

*ATCC* American Type Culture Collection, *ESR* Institute of Environmental Science and Research Limited, *UoA* University of Auckland.

Pure cultures of each species were prepared according to established laboratory protocols. Briefly, *B. fragilis* and *F. nucleatum* lyophilized cells were rehydrated with cooked meat medium then plated onto sheep blood agar and incubated at 37 °C under anaerobic conditions. Liquid broth cultures of *B. fragilis* and *F. nucleatum* were also prepared in cooked meat medium and incubated at 37 °C under static, anaerobic conditions. *H. influenzae* lyophilized cells were rehydrated using brain heart infusion (BHI) medium then plated onto supplemented chocolate agar and incubated at 37 °C and 5% CO_2_ conditions. *S. pyogenes* and *P. aeruginosa* were cultured from frozen stocks into BHI broth and grown overnight at 37 °C under static conditions or 200 rpm, respectively. *E. coli* was cultured from frozen stocks into Luria Bertani broth overnight at 37 °C, 200 rpm.

Growth on agar and broth was assessed after 12 h, 24 h, and 48 h. For *B. fragilis, F. nucleatum, P. aeruginosa, S. pyogenes* and *E. coli*, the broth cultures were centrifuged at 4,500 rpm for 10 min and the supernatant was discarded. For *H. influenzae*, turbid growth in BHI broth was not observed, so colonies from the supplemented chocolate agar were used. All cultures were resuspended in 1 mL of 1X PBS then centrifuged at 15,000×*g*. The supernatant was discarded, and the pellets were resuspended in 500 µL of PFA and incubated for 3 h at room temperature. After PFA incubation, bacterial pellets were washed twice with 1 mL of 1X PBS before resuspending in 50:50 100% ethanol and Tris-buffered Saline (TBS).

### FISH on pure bacterial cell cultures

When determining the sensitivity of each FISH probe, standard concentrations of 20–50 µg/ml were initially used and subsequently optimised to provide a positive signal^18^. The incubation chamber was set to 48 °C. Bacterial isolates were removed from the − 20 °C freezer and were set to thaw on ice. A medium-sized circle was drawn on a Superfrost ® Plus Positively Charged Microscope Slide using a PAP hydrophobic pen (Abcam 2601, Cambridge, UK). The bacterial isolates were mixed for 10 s, before pipetting 60 µL of an individual bacterial isolate within the drawn circle and the slide being placed in the incubation chamber for 20 min. Once dry, each slide was then dehydrated in ascending ethanol concentrations (50%, 86% and 96%) sequentially, for 3 min each. Fresh hybridisation buffer was then made, containing 180 µL of 5 M sodium chloride (NaCl), 20 µL of 1 M Tris aminomethane hydrochloride (Tris/HCl), 799 µL of ddH_2_0 (Sartorius Stedim Biotech Filtration System, Göttingen, Germany) and 1 µL of 10% sodium dodecyl sulphate (SDS) per 1 mL. Following dehydration in 100% ethanol, the slides were air-dried for 1 min, while FISH probe solutions were made using hybridisation buffer as the diluent. Once dry, the slides were then placed in an opaque plastic tray, and 60 µL of probe solution was pipetted onto the isolates. Residual hybridisation buffer was poured onto the base of the tray to minimise the risk of slides drying out during the incubation period. The plastic trays were then sealed with masking tape before undergoing hybridisation at 48 °C for 5 h in darkness. The FISH protocol used was adapted from Neugent et al.^19^.

Fresh wash buffer (72 mL of 5 M NaCl, 8 mL of 1 M Tris/HCl, 320 mL of ddH_2_O and 400 µL of 10% SDS) was made and placed in a 46 °C water bath for 30 min. Finally, 400 mL of ddH_2_O was left to chill in a − 20 °C freezer. Upon completion of the hybridisation period, the lab lights were dimmed, and all slides were visualised to ensure hybridisation solution did not evaporate; sections without solution were recorded. Slides were then stacked inside the wash buffer with enough buffer solution to cover each slide. The slides and wash buffer were placed back into the water bath for 30 min in a dark room. Following washing, each slide was rinsed in the pre-chilled ddH_2_O to remove excess salts, before being dried in the incubation chamber at 48 °C for 10 min. Two hundred µL of 1:10,000 DAPI was then added to each slide and left to sit at room temperature for 20 min in darkness. Slides were rinsed again in chilled ddH_2_O and dried in the incubation chamber at 48 °C degrees for 10 min. Once completely dry, each slide was mounted with Citifluor Antifadent solution (AF × 1, Agar Scientific, Essex, UK) and a Trajan Series 1 Coverslip (Trajan Scientific and Medical, Germany) was added.

### Specificity of single FISH probes on mixed bacterial cell cultures

The specificity of the FISH probe was also tested using the protocol described in “FISH on pure bacterial cell cultures”, with a few minor alterations. The total volume of bacteria pipetted on each slide remained 60 µL but was divided to contain equal proportions of the chosen isolates. For example, when testing the STRC493 probe on mixed bacterial cell culture of *S. pyogenes* and *E. coli*, 30 µL of *S. pyogenes* and 30 µL of *E. coli* (50:50) were combined, mixed and pipetted onto the slide.

Initially, each FISH probe was tested against the probe’s matching bacterial isolate and *E. coli*. If specific, it was then tested against additional species of similar morphology and proximity on the phylogenetic tree, to further ensure specificity. This work was performed in addition to previously published specificity analyses of these probes, as is listed in Table A1. in the Supplementary Information^11–16, 20^.

### Multiple specific FISH probes in mixed bacterial cell cultures

Multiple probes were combined, using different carbocyanine dyes at the 5’ end of the probe, to identify multiple bacterial taxa on the same slide. One probe had a Cy3 fluorescent dye (greenish-yellow region, ~ 550 nm excitation, ~ 570 nm emission), and the other had a Cy5 fluorescent dye (red region, ~ 650 nm excitation, ~ 670 nm emission), in order to distinguish between the two probes simultaneously. The universal eubacterial probe (EUB338) consistently had a fluorescein isothiocyanate (FITC) fluorescent dye only. All probes then underwent the protocol outlined in “Specificity of single FISH probes on mixed bacterial cell cultures”.

### Tissue sample collection and preparation for molecular analysis

Bilateral palatine tonsils were collected from 24 patients undergoing extracapsular tonsillectomy for recurrent tonsillitis (RT) (n = 14) or sleep-disordered breathing (SDB) (n = 10) as part of a previous study^3^. FISH was not performed in this earlier study, and only new data concerning the optimisation of bacterial FISH probes on the remaining tissue are published here. Ethical approval was received by the Health and Disability Ethics Committee (HDEC) of New Zealand, (Ethics number 16/STH/53) and written informed consent was obtained from all caregivers preoperatively. All methods were performed in accordance with HDEC guidelines and regulations. All procedures were performed under general anaesthetic, by a single surgeon, and using either coblation or bipolar diathermy.

Immediately following resection, the left and right tonsil specimens were placed into two individual sterile containers and labelled accordingly. Both tonsils were then stored on ice and returned to the lab within 2 h after resection. Once returned to the lab, the left palatine tonsils were fixed in Carnoy’s, before being embedded in paraffin wax blocks using standard techniques^21^. The right tonsils were not used in this study. All specimens used in this study were fixed, as this is the standard protocol for preserving tissue specimens for histological analysis in our laboratory. Use of a fixative agent also enabled simultaneous analysis of both histological markers and bacterial cells in a single specimen. Tissue sections of each palatine tonsil in the coronal plane were sectioned at intervals of 250 μm. Five adjacent sections were cut at each point, all at a thickness of 5 μm, and mounted on Superfrost® Plus Positively Charged Microscope Slides (Thermo Fisher Scientific, Auckland, New Zealand).

### Histological analysis

#### Gram staining

A Gram stain was performed on the first section of each 250 μm interval for each patient using standard protocols. In brief, each section was flooded with filtered crystal violet for 3 min, before being washed and flooded in Lugol’s iodine for 3 min. Acetone was then used to decolourise each section, before being rinsed in tap water and counterstained with 1% safranin. Each slide was then rinsed in tap water, before being dehydrated in 100% ethanol twice, 5 min each time. Each slide was immersed in xylene twice, 5 min each time. Finally, slides were mounted with dibutylphthalate polystyrene xylene (DPX) (Scharlau Barcelona, Spain), and a Trajan Series 1 Coverslip (Trajan Scientific and Medical, Germany) was added. Each section was then screened for the presence of bacterial microcolonies.

#### Imaging

Each Gram stained section was examined using bright field light microscopy with a Leica DMR upright microscope (Leica Microsystems, Wetzlar, Germany). Photographs were taken with a Nikon Digital Sight DS-5Mc-U1 cooled colour camera (Nikon, Tokyo, Japan), using Nikon NIS Elements for Image Acquisition software (Nikon, Tokyo, Japan). All sections were screened at 10X magnification and examined for the presence of bacterial microcolonies.

### Application of bacterial FISH probes in tonsil sections

Each probe was then applied to a section of tonsil tissue using the protocol outlined in 2.3, with a few amendments. First, each tonsil section was deparaffinized twice in xylene for 5 min, before dehydration in ascending ethanol concentrations (50%, 86% and 96%) sequentially, for 3 min each time. Second, the volume of the probe solution and DAPI were increased to 200 µL for each tonsil section. Finally, positive controls using pure bacterial isolates were performed alongside tonsil sections for each FISH probe, to ensure the accuracy of the protocol. Once protocols were optimised for individual probes in tonsil sections, multiple probes were then applied to tonsil tissue.

### Fluorescent imaging and analysis

All slides were examined under fluorescence microscopy using the Olympus FV1000 Confocal Laser Scanning Microscope (Olympus Corporation, Tokyo, Japan), FluoView Software (version 4.2) (Olympus Corporation, Tokyo, Japan). Each tissue section was first scanned for microcolonies, prior to analysing the presence and location of bacterial probe staining. Microcolonies were defined, then considered eligible for inclusion in downstream analyses using the following criteria: an (i) aggregation of bacteria, that is (ii) encased by a complex extracellular matrix, and is (iii) embedded in tissue (e.g. not artifact on slide)^22–24^. Positive bacterial isolate culture controls were always examined and imaged alongside the tissue.

An image of each slide was taken, with nuclei/DAPI labelled in blue, the universal eubacterial probe in green, the first bacteria specific probe in red and the second bacteria specific probe in yellow (if present). An image of each eligible microcolony identified was also captured, using consistent colour coding. All images were obtained using a × 10 objective, at 2048 × 2048 pixels and with a zoom of 2 (Nyquist = 2.3). Semi-automated analysis using ImageJ was used to investigate the prevalence of different bacterial taxa within tonsillar microcolonies. Prevalence was defined as the percentage of a bacterial specific probe within a bacterial microcolony. First, the area of each whole microcolony was calculated, using the FITC EUB probe. Next, the area of the Cy3 bacteria specific probe within the defined perimeter of the entire microcolony was calculated. The prevalence was then expressed as a percentage of the total bacteria in the image. Individual bacteria specific probes were used to calculate area, as opposed to composite images, in order to minimise the risk of incorrect analysis due to colour distortion or inaccurate readings.

### Statistical analysis

Univariate analysis was applied to analyse potential patient factors that were associated with differences between patients with RT and SDB. Categorical variables were assessed using *Chi*-square tests. Student’s T-tests were performed to analyse continuous variables. A two-tailed *p*-value of less than 0.05 was considered statistically significant. All statistical analyses were performed using *Wizard Pro* software (version 1.9.32).

## Results

### FISH on pure bacterial isolates

**Figure 1:**
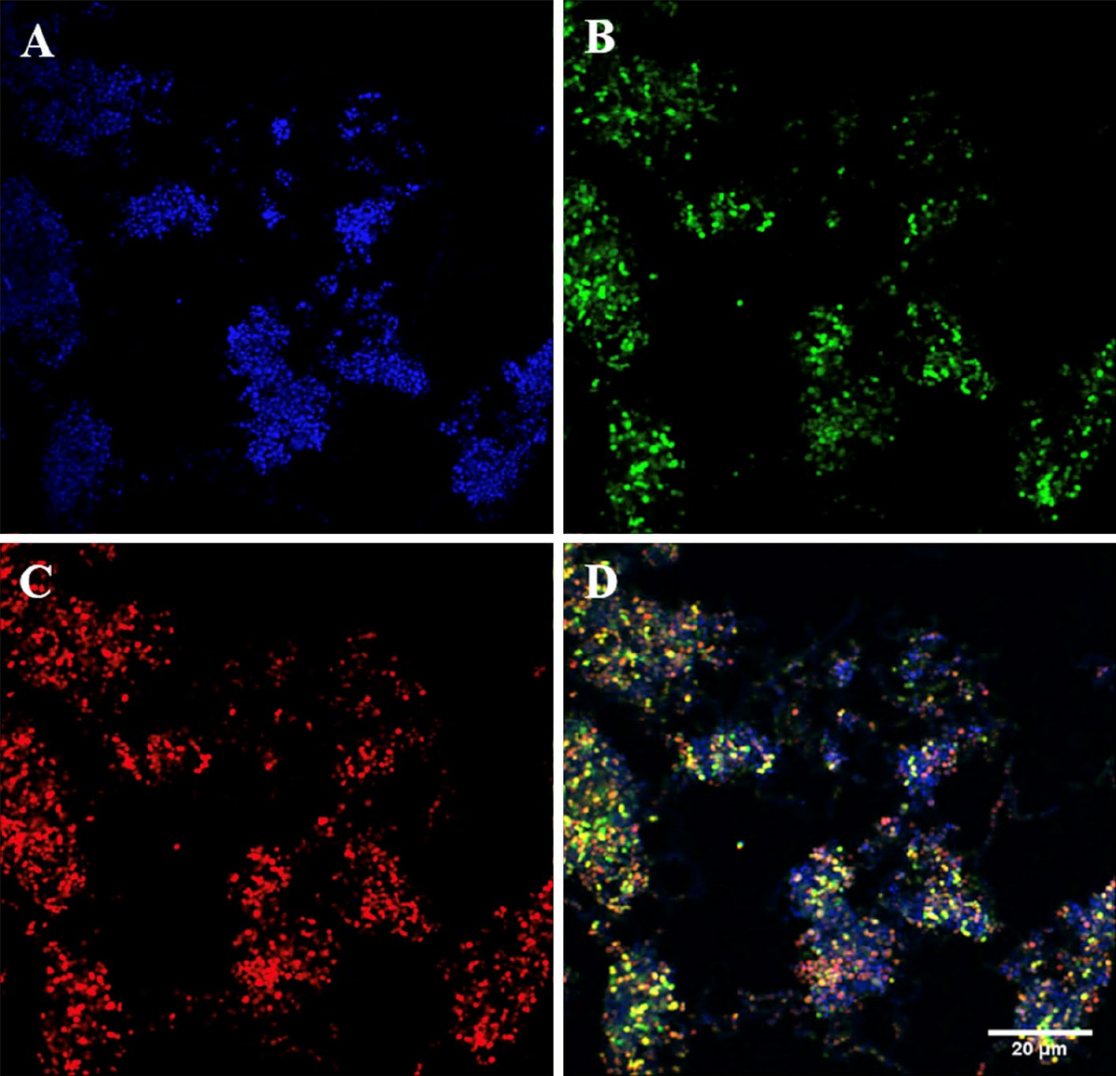
Fluorescence in situ hybridisation staining of pure *Streptococcus pyogenes* ATCC 700294 cells from broth culture. (**A**) DAPI in blue identifying all of the nucleic material present, (**B**) Eubacterial probe (EUB) in green identifying all of the bacterial present, (**C**) *Streptococcus* spp*.* specific probe (STRC493) in red highlighting all of the *Streptococcus* spp. present, (**D**) Composite image of figure (**A**–**C**). Images at ×60 magnification.

The sensitivity of five fluorescent oligonucleotide probes were all successfully optimised using their corresponding bacterial isolate. This included *Bacteroides* spp*.*, *Fusobacterium* spp*.*, *Streptococcus* spp*.*, *H. influenzae* and *Pseudomonas* spp. An image from the optimised protocol for the *Streptococcus* spp*.* probe in pure *S. pyogenes* culture is illustrated in Fig. 1. Representative images of the remaining probes with their corresponding pure bacterial isolates are included in the supplementary (Fig. A1–A4).

### Specificity of single FISH probes in mixed bacterial culture

**Figure 2:**
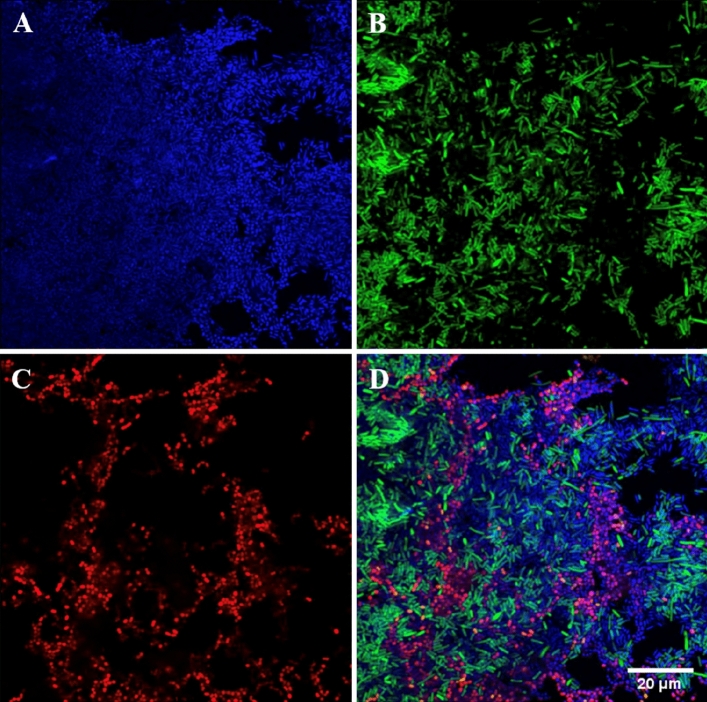
Fluorescence in situ hybridisation staining of *Streptococcus pyogenes* ATCC 700,294 and *Escherichia coli* K-12 DH5 alpha, ATCC PTA-4079 mixed cells from broth culture. (**A**) Nucleic material/DAPI in blue, (**B**) Eubacterial probe (EUB) in green, (**C**) *Streptococcus* spp*.* specific probe (STRC493) in red, (**D**) composite image of (**A**–**C**). Images at ×60 magnification.

The specificity of the five sensitive fluorescent oligonucleotide probes in mixed species cultures was all successfully optimised. A representative image from the optimised protocol for the *Streptococcus* spp*.* probe in mixed bacterial isolate culture is illustrated in Fig. 2. Images of the remaining probes with their corresponding mixed bacterial isolates are included in the supplementary (Fig. A5–A8).

### Multiple taxa specific FISH probes in mixed bacterial isolate culture

**Figure 3:**
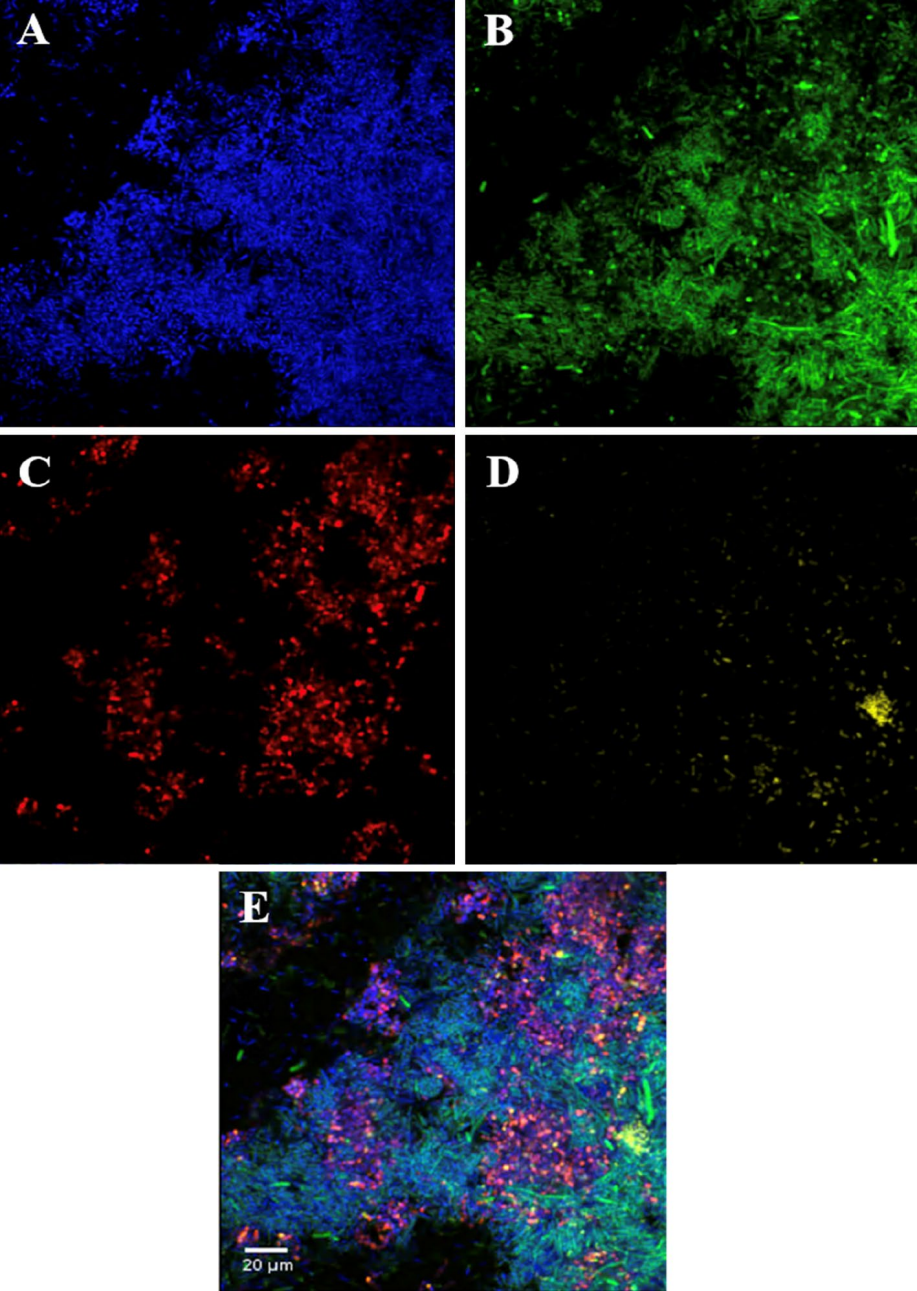
Fluorescence in situ hybridisation staining of *Streptococcus pyogenes* ATCC 700,294, *Pseudomonas aeruginosa* ATCC BAA-47 and *Escherichia coli* K-12 DH5 alpha, ATCC PTA-4079 mixed cells from broth culture. (**A**) Nucleic material/DAPI in blue, (**B**) Eubacterial probe (EUB) in green, (**C**) *Streptococcus* spp*.* specific probe (STRC493) in red, (**D**) *Pseudomonas* spp*.* specific probe (PSE227) in yellow, (**E**) composite image of (**A**–**D**). Images at ×60 magnification.

Using our protocol, two taxa specific probes were successfully combined using different carbocyanine dyes (Cy3 and Cy5) at the 5’ end of the probes. These probes were applied using a combination of the two respective bacterial isolates (Fig. 3).

### Prevalence and characteristics of bacterial microcolonies in tonsil tissue

**Figure 4:**
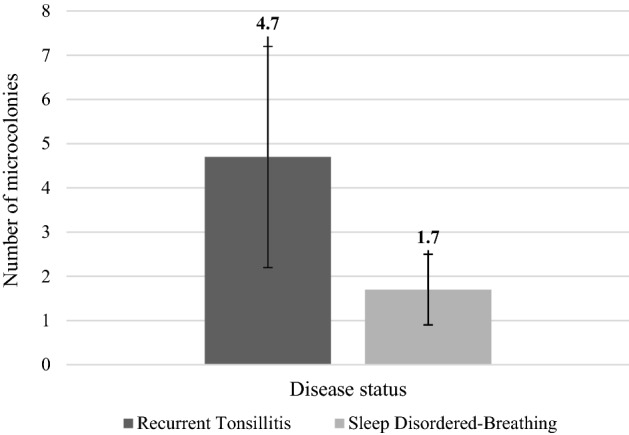
Number of bacterial microcolonies found per patient according to diagnosis. In total, 344 microcolonies were observed in 14 recurrent tonsillitis patients (4.7 ± SD 2.5 on average per person). In total, 24 microcolonies were observed in 10 sleep disordered-breathing patients (1.7 ± SD 0.8 per person) (*p* = 0.04).

From the 120 tonsil sections examined, 363 bacterial microcolonies were identified and imaged using confocal laser scanning microscopy. The majority of patients were male (54.2%). There were significantly more females in the RT group (64.3%) when compared to the SDB group (20.0%) (*p* = 0.03). Microcolonies were present in 91.7% of patients (22 out of 24). There were significantly more microcolonies in patients with RT (4.7 ± 2.5) when compared to patients with SDB (1.7 ± 0.8) (*p* = 0.04) (Fig. 4). The majority of patients had multiple microcolonies in a single section of tissue (11 RT patients and 5 SDB patients). Females had significantly more microcolonies per section of tissue (5.2 ± 3.0) when compared to males (2.0 ± 1.1) (*p* = 0.03). There were no significant differences in the average number of microcolonies present based on ethnicity, tonsil grade, history of asthma, number of courses of antibiotics prescribed in the preoperative year or positive GAS throat swab preoperatively (all *p* > 0.05).

The majority of microcolonies (97.8%, n = 355) were located in the tonsillar crypts. The average total area of the microcolonies identified was 54,544.4 ± 33,734.3 μm^2^. There were no significant differences in the average total area of microcolonies between the RT group (69,427.2 ± 46,410.5 μm^2^) and the SDB group (33,047.1 ± 56,84.0 μm^2^) (*p* > 0.05). No clinical or demographic variables were significantly associated with the average total area of microcolonies.

### FISH on bacterial microcolonies in tonsil tissue sections

**Figure 5:**
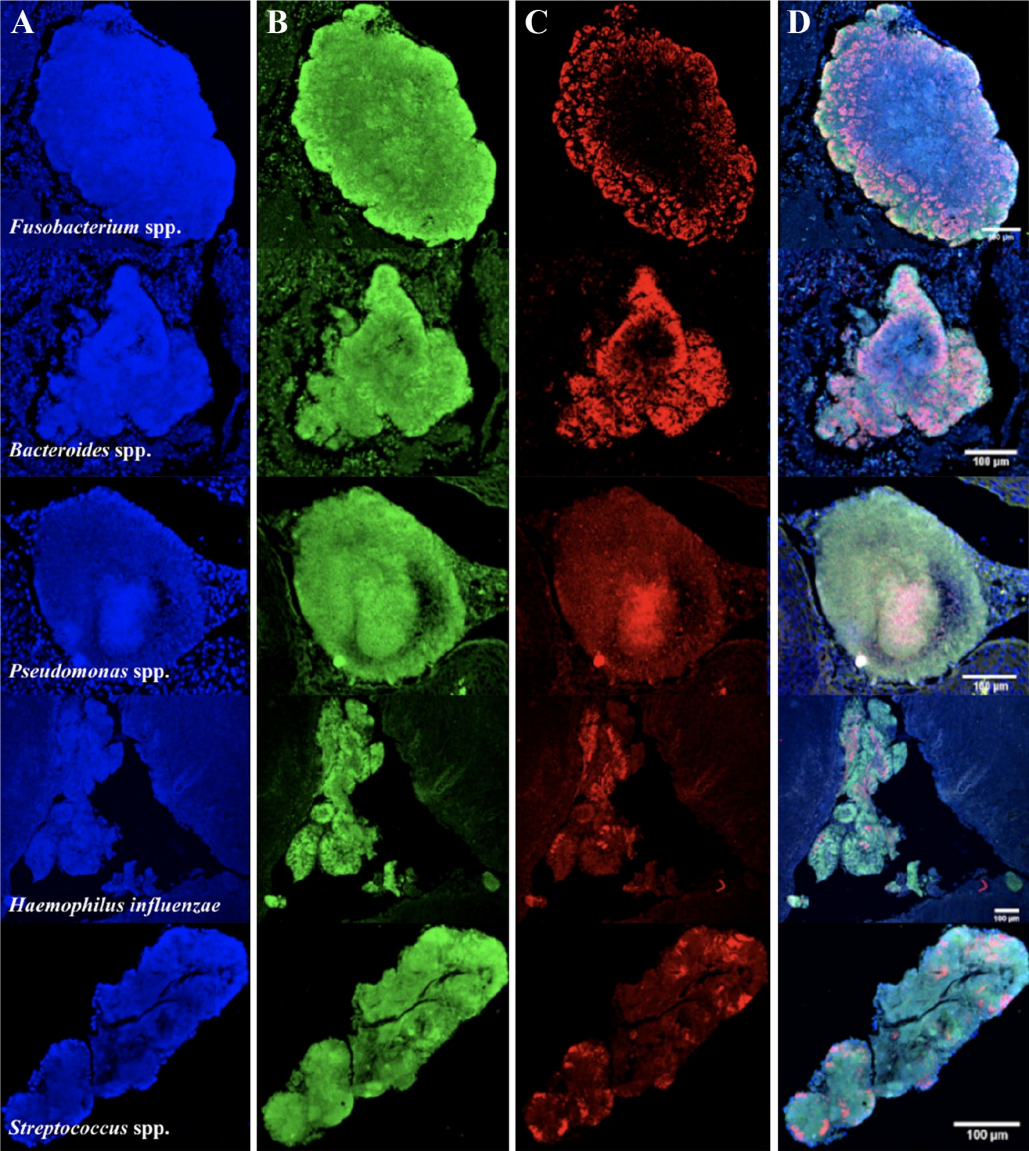
Fluorescence in situ hybridisation staining of *Fusobacterium* spp.*, Bacteroides* spp.*, Pseudomonas* spp.*, Haemophilus influenzae* and *Streptococcus* spp. probes in different bacterial microcolonies. Rows: *Fusobacterium* spp., *Bacteroides* spp., *Pseudomonas* spp., *H. influenzae* and *Streptococcus* spp. specific probes. Columns: (**A**) Nucleic material/DAPI in blue, (**B**) Eubacterial probe (EUB) in green, (**C**) Specific bacterial taxa probe in red, (**D**) composite image of (**A**–**C**). Images at ×10 magnification.

Five bacterial FISH probes were successfully optimised in Carnoy’s fixed tonsil tissue, as illustrated in Fig. 5. *Fusobacterium* spp. and *Bacteroides* spp. were located in the outer perimeter of the bacterial microcolonies identified. *Fusobacterium* spp*.* and *Bacteroides* spp*.* were also the most abundant of the bacterial probes in these microcolonies, when using semi-automated analysis. In contrast, *Pseudomonas* spp. and *H. influenza* were located towards the centre of the bacterial microcolonies identified. Finally, *Streptococcus* spp. were located in small peripheral clusters throughout the bacterial microcolonies. All bacterial-isolate controls were positive, and demonstrated findings consistent with Fig. 1 and Fig. A1–A4 (located in the Supplementary Material) for each of the bacterial probes, respectively. Representative images of all 5 probes within the same microcolony are also illustrated below in Figure A9 in the Appendix.

### Percentage area of bacteria specific probes within microcolonies

Semi-automated analysis using ImageJ revealed significant differences in the prevalence and the average percentage area of the bacterial FISH probes within tonsillar microcolonies. *Bacteroides* spp. were present in 100% of patients in which microcolonies were identified (22/22 patients with microcolonies, 57/363 microcolonies). In comparison, the prevalence of *Fusobacterium* spp. was 93.8%, *Streptococcus* spp. 85.7%, *H. influenzae* 82.35% and *Pseudomonas* spp. 76.5% in patients in which microcolonies were identified. There were no significant differences in the prevalence of any of the bacterial FISH probes analysed between disease groups. Overall, *Bacteroides* spp. had the highest average percentage area of 33.6 ± 14.8% within a microcolony and was closely followed by *Fusobacterium* spp. with an average percentage area of 26.3 ± 7.9%. In comparison, *Streptococcus* spp., *H. influenzae* and *Pseudomonas* spp., had average percentage areas of 10.5 ± 8.1%, 0.6 ± 0.8% and 0.4 ± 0.4%, respectively.

Statistically significant differences in the average percentage area of *Fusobacterium* spp. between disease groups (RT 31.9% ± 9.6 and SDB 14.0% ± 6.3; *p* = 0.02) were found. The difference in the average percentage area of *Bacteroides* spp. approached significance (RT 40.3 ± 16.7% and SDB 9.2 ± 19.7%; *p* = 0.06). There were no significant differences in the average percentage area of *Streptococcus* spp., *H. influenza.,* and *Pseudomonas* spp. between RT and SDB patients (all *p* > 0.05).

### Multiple taxa-specific FISH probes in tonsil tissue

**Figure 6:**
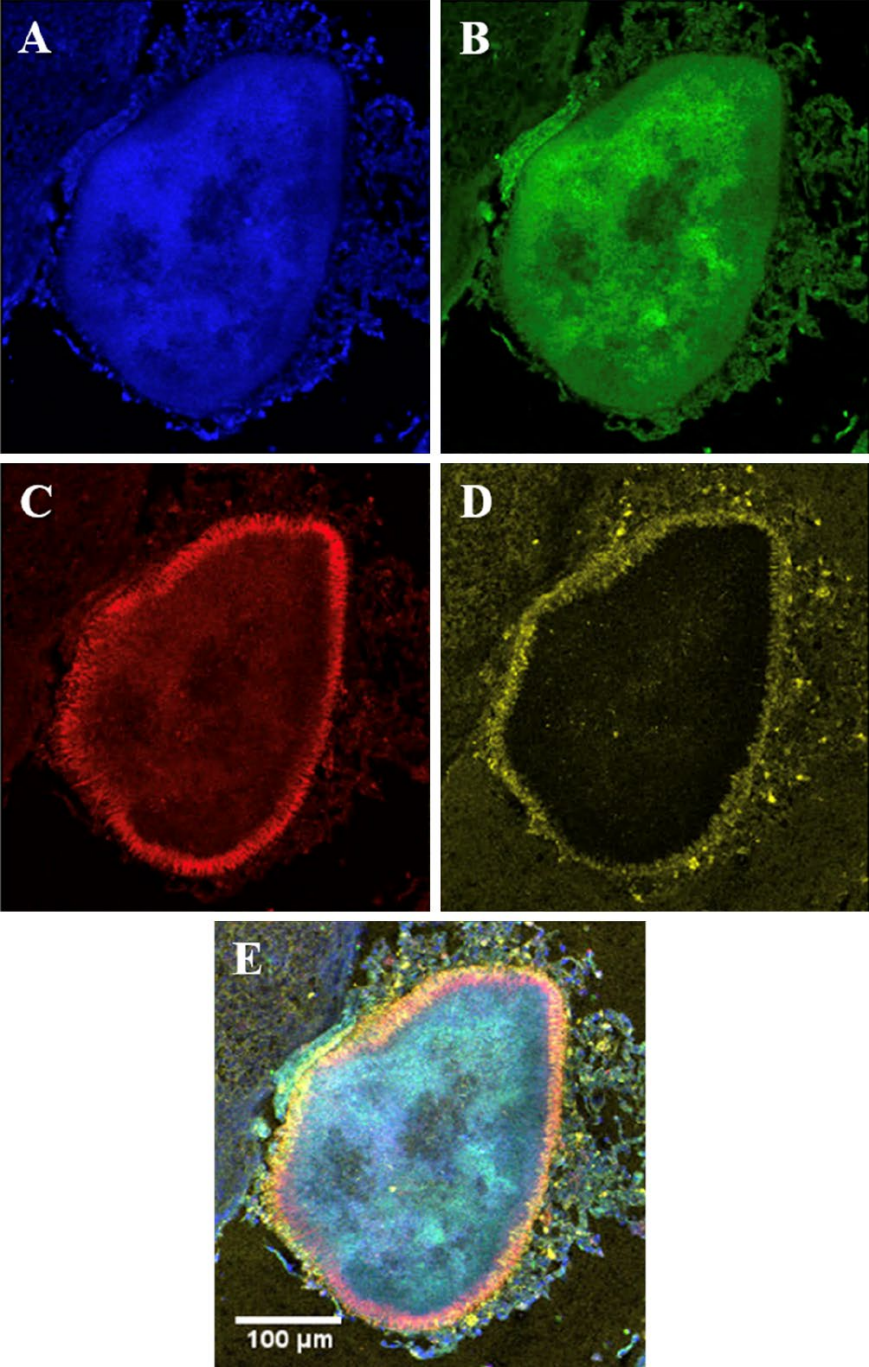
Fluorescence in situ hybridisation staining of *Fusobacterium* spp*.* and *Bacteroides* spp*.* in a single bacterial microcolony in tonsil tissue. (**A**) Nucleic material/DAPI in blue, (**B**) Eubacterial probe (EUB) in green, (**C**) *Fusobacterium* spp*.* specific probe (FUSO) in red, (**D**) *Bacteroides* spp*.* specific probe (BAC303) in yellow, (**E**) composite image of (**A**–**D**). Images at ×10 magnification.

Multiple taxa-specific probes were successfully applied simultaneously to the same section of tissue. In one microcolony, *Fusobacterium* spp*.* and *Bacteroides* spp*.* both distinctly located around the periphery of the microcolony, with some speckles of each bacterial probe visible towards the centre (Fig. 6).

## Discussion

The FISH protocol developed in this study was used to make novel insights into the composition and arrangement of common bacteria within microcolonies in TH. This study successfully optimised five FISH probes in vitro in pure bacterial isolates and in situ in Carnoy’s fixed tonsil tissue specimens. Besides Nistico et al., this is the first FISH methods protocol specific to tonsil tissue published in the literature in the last decade^1^. Critically, unlike previous studies implementing FISH in tonsil tissue, this is the first protocol tailored to Carnoy’s fixed specimens and bacterial microcolonies. Carnoy’s fixative preserves the tonsillar architecture for further spatial analysis and comparisons. Importantly, this preparation method allows for additional histological analysis of the same tissue section, reducing the number of specimens, time and resources required for combined histological and molecular analysis of specimens. Finally, this method protocol may also be adapted and applied to other human tissues fixed in the same manner.

### FISH confirmed the prevalence of anaerobic bacteria in tonsillar microcolonies

All five probes were detected within the microcolonies in the tissue sections examined. We observed notable trends in the organisation of these five probes within the microcolonies. *Streptococcus* spp*.*, *Haemophilus influenzae* and *Pseudomonas* spp*.* were primarily observed towards the centre of a microcolony and appeared to be less abundant. In comparison, *Fusobacterium* spp*.* and *Bacteroides* spp*.* were both observed towards the periphery of the microcolony and were the most abundant bacterial-species present within the microcolonies according to area analyses when using semi-automated analysis. Anaerobic bacteria have been rarely studied in the context of tonsillar hyperplasia^25^ and are typically identified in low quantities when using cultivation techniques^26^. Recent molecular based studies have identified increased quantities of anaerobic bacteria, including *Bacteroides* spp., *Fusobacterium* spp., *Prevotella* spp. and *Pseudomonas* spp. in the crypts of tonsils in children with TH. Our novel finding of an increased prevalence overall, and the peripheral arrangement of anaerobic species within microcolonies is unexpected given their metabolic requirements. The interactions between the various pathogens identified within these microcolonies remains unknown. These findings have led to an appreciation for the complex polymicrobial community in the tonsils of children with TH. Understanding the bacterial and immunological interactions within the tonsillar crypts is a key factor for deciphering the pathogenesis of TH.

### Comparisons of sampling methodologies in tonsillar hyperplasia

FISH has proven to be an effective method of identifying and quantifying both aerobic and anaerobic bacteria in tonsil tissue. It is also unique in its ability to provide a two-dimensional visualisation of the microorganisms in situ^1^. Culture-dependent techniques such as tonsil swabs are unable to detect the presence of microcolonies in the tonsillar crypts. They are also unable to sample the bacterial communities within the microcolonies^2, 8, 27^. In comparison, molecular techniques such as FISH allow for specific analysis of bacterial microcolonies, while still preserving the surrounding environment. These in situ analyses allow for the investigation of intracellular bacteria located within the tonsil parenchyma, which have also been implicated in the pathogenesis of TH^8^.

Similarly, diagnostic laboratories typically do not perform extensive culturing beyond standard pathogens such as *Streptococcus* spp*.* or *Fusobacterium* spp. Traditional culture techniques are often limited in their ability to cultivate results that accurately reflect a diverse microbial community, as is the case with TH^26, 28–30^. The ability of these culture-based studies to quantify differences in the bacteria present is also heavily dependent on the media and laboratory environment used^21, 31^. Subsequently, traditional culture-based studies investigating the microbiome associated with TH provide a narrow insight into the pathogens driving the disease. These findings emphasise the importance of using culture-independent techniques to accurately identify and quantify a diverse microbial community, as seen in TH. Furthermore, the ability of molecular techniques to identify organisms not easily cultured is particularly important, considering the abundance of anaerobic bacteria identified. These advantages were demonstrated in a recent study by Johnston et al., which utilised 16 s rRNA gene sequencing to investigate the bacterial community composition of tonsillar crypts in patients with RT and OSA^3^. Despite this, much of current clinical practice and decision making is based on the findings of these culture based studies. The findings of this study are in keeping with a growing body of evidence that supports a polymicrobial community that is exposed to various aerobic-anaerobic environments on the surface and crypts of tonsils^21, 32–34^. However, due to the ethical limitations of surgically resecting healthy tonsils from patients, our protocol was unable to be performed on healthy controls for comparative analysis. This remains an inherent limitation of tonsillar microbiome research. Finally, the described methodology in this protocol provides a strong foundation for future research in this area and in other human tissue samples prepared using the same techniques.

### Future directions

Future work may involve optimising additional FISH probes targeting a broad range of specific bacterial species which have been implicated in the pathogenesis of TH. Other potential pathogens of interest include bacteria from the genera *Treponema*^3, 35, 36^, *Actinomyces*^37^, *Staphylococcus*^38^*, Parvimonas*, *Prevotella, Porphyromonas, Neisseria, Moraxella*^3, 39, 40^*, Capnocytophaga* and *Corynebacterium*^41^. This would allow for a more comprehensive analysis of the composition and arrangement of bacterial species within microcolonies. The detailed methods outlined in this study provide a robust foundation and protocol for optimising additional FISH probes in the future.

The relationship between different bacterial species, archaea, fungi and viruses within tonsillar microcolonies also remains unknown. Multiple taxa-specific FISH probes with different fluorescent tags can be applied to the same tonsil section allowing for the detection of multiple pathogens at an identical point in time within the same microcolony. Importantly, this would also allow for any co-localization between different pathogens to be detected. This would also enable further investigation into the host–pathogen interaction in paediatric TH. Finally, combining these FISH protocols with other molecular techniques such as quantitative PCR and laser microdissection (LMD) in future experiments may allow for a more accurate measurement of the abundance and arrangement of taxa-specific species bacteria within tonsil tissue.

## Conclusion

The FISH protocol developed in this study for Carnoy’s fixed tonsil tissue specimens identified both aerobic and anaerobic bacteria within tonsillar microcolonies. These results have provided novel insights into the composition and arrangement of common bacteria within microcolonies in TH, and emphasise the importance of using culture-independent techniques such as FISH to accurately identify and quantify a diverse microbial community, as seen in TH. Finally, the described methodology in this study provides a strong foundation for future microbiological and immunohistochemical research in both TH and other disease states.

## Supplementary Information

Supplementary Information.

## Data Availability

The datasets used and/or analysed during the current study are not publicly available as they primarily consist of images, but may be made available from the corresponding author on reasonable request.

## References

[1] Nistico L, Sokolowski B (2009). Fluorescence “*in situ*” hybridization for the detection of biofilm in the middle ear and upper respiratory tract mucosa. Auditory and Vestibular Research: Methods and Protocols.

[2] Swidsinski A (2007). Spatial organisation of microbiota in quiescent adenoiditis and tonsillitis. J. Clin. Pathol..

[3] Johnston J (2018). The bacterial community and local lymphocyte response are markedly different in patients with recurrent tonsillitis compared to obstructive sleep apnoea. Int. J. Pediatr. Otorhinolaryngol..

[4] Heiniger N, Spaniol V, Troller R, Vischer M, Aebi C (2007). A reservoir of *Moraxella catarrhalis* in human pharyngeal lymphoid tissue. J. Infect. Dis..

[5] Hoa M (2009). Identification of adenoid biofilms with middle ear pathogens in otitis-prone children utilizing SEM and FISH. Int. J. Pediatr. Otorhinolaryngol..

[6] Nistico L (2011). Adenoid reservoir for pathogenic biofilm bacteria. J. Clin. Microbiol..

[7] Zautner AE (2010). Intracellular persisting *Staphylococcus aureus* is the major pathogen in recurrent tonsillitis. PLoS ONE.

[8] Stepinska M, Olszewska-Sosinska O, Lau-Dworak M, Zielnik-Jurkiewicz B, Trafny EA (2014). Identification of intracellular bacteria in adenoid and tonsil tissue specimens: The efficiency of culture versus fluorescent *in situ* hybridization (FISH). Curr. Microbiol..

[9] Chen R (2022). The histological and microbiological characteristics of bacterial microcolonies in paediatric tonsillar hyperplasia. Int. J. Pediatr. Otorhinolaryngol..

[10] Greuter, D., Loy, A., Horn, M. & Rattei, T. List of probes tested for in situ hybridization. *probeBase* http://probebase.csb.univie.ac.at/pb_results/listinsitu/0 (2016).

[11] Manz W, Amann R, Ludwig W, Vancanneyt M, Schleifer KH (1996). Application of a suite of 16S rRNA-specific oligonucleotide probes designed to investigate bacteria of the phylum *cytophaga-flavobacter-bacteroides* in the natural environment. Microbiology.

[12] Sunde PT (2003). Fluorescence *in situ* hybridization (FISH) for direct visualization of bacteria in periapical lesions of asymptomatic root-filled teeth. Microbiology.

[13] Franks AH (1998). Variations of bacterial populations in human feces measured by fluorescent *in situ* hybridization with group-specific 16S rRNA-targeted oligonucleotide probes. Appl. Environ. Microbiol..

[14] Watt M, Hugenholtz P, White R, Vinall K (2006). Numbers and locations of native bacteria on field-grown wheat roots quantified by fluorescence *in situ* hybridization (FISH). Environ. Microbiol..

[15] Hogardt M (2000). Specific and rapid detection by fluorescent in situ hybridization of bacteria in clinical samples obtained from cystic fibrosis patients. J. Clin. Microbiol..

[16] Amann RI (1990). Combination of 16S rRNA-targeted oligonucleotide probes with flow cytometry for analyzing mixed microbial populations. Appl. Environ. Microbiol..

[17] Wagner Mackenzie B (2021). Characterising clinical *Staphylococcus aureus* isolates from the sinuses of patients with chronic rhinosinusitis. Sci. Rep..

[18] Liehr T, Kreskowski K, Ziegler M, Piaszinski K, Rittscher K, Liehr T (2017). The standard FISH procedure. Fluorescence In Situ Hybridization (FISH): Application Guide.

[19] Neugent ML, Gadhvi J, Palmer KL, Zimmern PE, De Nisco NJ (2019). Detection of tissue-resident bacteria in bladder biopsies by 16S rRNA fluorescence in situ hybridization. J. Vis. Exp..

[20] Kempf VA, Trebesius K, Autenrieth IB (2000). Fluorescent in situ hybridization allows rapid identification of microorganisms in blood cultures. J. Clin. Microbiol..

[21] Sarkar S, Sil A, Sarkar S, Sikder B (2017). A comparison of tonsillar surface swabbing, fine-needle aspiration core sampling, and dissected tonsillar core biopsy culture in children with recurrent tonsillitis. Ear Nose Throat J..

[22] Costerton JW, Stewart PS, Greenberg EP (1999). Bacterial biofilms: a common cause of persistent infections. Science.

[23] Hall-Stoodley L, Stoodley P (2009). Evolving concepts in biofilm infections. Cell. Microbiol..

[24] Parsek MR, Singh PK (2003). Bacterial biofilms: An emerging link to disease pathogenesis. Annu. Rev. Microbiol..

[25] Kalaiarasi R, Subramanian KS, Vijayakumar C, Venkataramanan R (2018). Microbiological profile of chronic tonsillitis in the pediatric age group. Cureus.

[26] Brook I, Yocum P, Foote PA (1995). Changes in the core tonsillar bacteriology of recurrent tonsillitis: 1977–1993. Clin. Infect. Dis..

[27] Johnston JJ, Douglas R (2018). Adenotonsillar microbiome: An update. Postgrad. Med. J..

[28] Gaffney RJ, Timon CI, Freeman DF, Walsh MA, Cafferkey MT (1993). Bacteriology of tonsil and adenoid and sampling techniques of adenoidal bacteriology. Respir. Med..

[29] Brook I, Yocum P (1984). Bacteriology of chronic tonsillitis in young adults. Arch. Otolaryngol..

[30] Timon CI, McAllister VA, Walsh M, Cafferkey MT (1990). Changes in tonsillar bacteriology of recurrent acute tonsillitis: 1980 vs. 1989. Respir. Med..

[31] Ren T (2013). 16 S rRNA survey revealed complex bacterial communities and evidence of bacterial interference on human adenoids. Environ. Microbiol..

[32] Dickinson A (2020). Tonsillar surface swab bacterial culture results differ from those of the tonsillar core in recurrent tonsillitis. Laryngoscope.

[33] Jensen A, Fago-Olsen H, Sorensen CH, Kilian M (2013). Molecular mapping to species level of the tonsillar crypt microbiota associated with health and recurrent tonsillitis. PLoS ONE.

[34] Wang Q (2017). Bacteriology and antibiotic sensitivity of tonsillar diseases in chinese children. Eur. Arch. Otorhinolaryngol..

[35] Seerangaiyan K, van Winkelhoff AJ, Harmsen HJM, Rossen JWA, Winkel EG (2017). The tongue microbiome in healthy subjects and patients with intra-oral halitosis. J. Breath Res..

[36] Watanabe H (2017). Comprehensive microbiome analysis of tonsillar crypts in IgA nephropathy. Nephrol. Dial. Transplant.

[37] Kutluhan A, Salvız M, Yalçıner G, Kandemir O, Yeşil C (2011). The role of the actinomyces in obstructive tonsillar hypertrophy and recurrent tonsillitis in pediatric population. Int. J. Pediatr. Otorhinolaryngol..

[38] Brook I, Yocum P, Shah K (1980). Surface vs core-tonsillar aerobic and anaerobic flora in recurrent tonsillitis. JAMA.

[39] Tejesvi MV (2016). Tonsillar microbiota in children with PFAPA (periodic fever, aphthous stomatitis, pharyngitis, and adenitis) syndrome. Eur. J. Clin. Microbiol. Infect. Dis..

[40] Brook I (2005). The role of anaerobic bacteria in tonsillitis. Int. J. Pediatr. Otorhinolaryngol..

[41] Batty A, Wren MWD (2005). Prevalence of *Fusobacterium necrophorum* and other upper respiratory tract pathogens isolated from throat swabs. Br. J. Biomed. Sci..

