# Supplementary material for: Fluorescence in situ hybridisation in Carnoy’s fixed tonsil tissue
Source: Sci Rep. 2022 Jul 20;12:12395. doi: 10.1038/s41598-022-16309-w (PMC9300673; doi:10.1038/s41598-022-16309-w)
Supplement: Supplementary file 1 — Supplementary Information. [file 41598_2022_16309_MOESM1_ESM.docx]

**Supplementary Information**

**Table A.1 Target organisms and reference stains used to test specificity of FISH probes.**

| **Organisms and Strains** | **Source*** | **Hybridization results from the following FISH probes** | | | | |
| --- | --- | --- | --- | --- | --- | --- |
|  |  | ***BAC303*** | ***FUSO*** | ***STRC493*** | ***HAEinf*** | ***PSE277*** |
|  |  | [*^9^*](https://paperpile.com/c/aYBYBa/jIkPV) | [*^10^*](https://paperpile.com/c/aYBYBa/08Xi5) | [*^11^*](https://paperpile.com/c/aYBYBa/2Ggvy) | [*^12^*](https://paperpile.com/c/aYBYBa/7ecHw) | [*^13^*](https://paperpile.com/c/aYBYBa/WqAmQ) |
| *Acinetobacter sp.* | RA3957 | - | - | - | - | N |
| *Actinobacillus actinomycetemcomitans* | ATCC 43718 | - | N | - | - | - |
| *Alcaligenes faecalis* | DSM 30030 | - | - | - | N | - |
| *Alcaligenes xylosoxidans* | CF (4×) | - | - | - | N | - |
| *Bacteroides distasonis* | DSM 20701 | - | - | N | - | - |
| *Bacteroides distasonis* | ATCC 8503^T^ | P | - | - | - | - |
| *Bacteroides eggerthii* | DSM 20697 | - | - | N | - | - |
| *Bacteroides eggerthii* | ATCC 27754 ^T^ | P | - | - | - | - |
| *Bacteroides fragilis* | DSM 2151 | - | - | N | - | - |
| *Bacteroides fragilis* | ATCC 25285 ^T^ | P | - | - | - | - |
| *Bacteroides fragilis* | DSM 1396 | - | - | - | - | - |
| *Bacteroides ovatus* | ATCC 8483 ^T^ | P | - | - | - | - |
| *Bacteroides thetaiotaomicron* | ATCC 2914g ^T^ | P | - | - | - | - |
| *Bacteroides vulgatus* | DSM 1447 | - | - | N | - | - |
| *Bacteroides vulgatus* | ATCC 8482 ^T^ | P | - | - | - | - |
| *Bergeyella zoohelcum* | LMG 8351 ^T^ | N | - | - | - | - |
| *Bergeyella zoohelcum* | LMG 8352 | N | - | - | - | - |
| *Bifidobacterium infantis* | ATCC 15697 | - | - | N | - | - |
| *Bordetella pertussis* | DSM 5571 | - | - | - | N | - |
| *Burkholderia andropogonis* | DSM 9511 | - | - | - | N | - |
| *Burkholderia caryophilli* | DSM 50341 | - | - | - | N | - |
| *Burkholderia cepacia* | ATCC 25416 | - | - | - | N | - |
| *Burkholderia cepacia* | CF (10×) | - | - | - | N | - |
| *Burkholderia gladioli* | DSM 4285 | - | - | - | N | - |
| *Burkholderia vietnamiensis* | LMG 10929 | - | - | - | N | - |
| *Campylobacter rectus* | ATCC 33238 ^T^ | - | N | - | - | - |
| *Candida glabrata* | ATCC 90030 | - | - | - | - | - |
| *Candida krusei* | ATCC 6258 | - | - | - | - | - |
| *Candida parapsilosis* | DSM 70125 | - | - | - | - | - |
| *Capnocytophaga gingivalis* | MCCM 00858 | - | N | - | - | - |
| *Carnobacterium divergens* | DSM 20623 | - | - | N | - | - |
| *Chryseobacterium gleum* | LMG 8334 | N | - | - | - | - |
| *Chyseobacterium indologenes* | LMG 8336 | N | - | - | - | - |
| *Chyseobacterium indologenes* | LMG 8337 ^T^ | N | - | - | - | - |
| *Citrobacter freundii* | ATCC 6750 | - | - | - | - | - |
| *Citrobacter freundii* | ATCC 8090 | - | - | - | - | - |
| *Clostridium bifermentans* | NIZO B529 | - | - | N | - | - |
| *Clostridium difficile* | ATCC 9688 | - | - | N | - | - |
| *Clostridium histolyticum* | DSM 2158 | - | - | N | - | - |
| *Clostridium oroticum* | DSM 1287 | - | - | N | - | - |
| *Cytophaga hutchinsonii* | LMG 10844 ^T^ | N | - | - | - | - |
| *Cytophaga johnsonae* | LMG 1341 ^T^ | N | - | - | - | - |
| *Cytophaga johnsonae* | LMG 1342 | N | - | - | - | - |
| *Cytophaga uliginosa* | LMG 3809 ^T^ | N | - | - | - | - |
| *Cytophaga xylanolytica* | DSM 6779 | - | - | N | - | - |
| *Empedobacter brevis* | LMG 4011 ^T^ | N | - | - | - | - |
| *Empedobacter brevis* | LMG 4012 | N | - | - | - | - |
| *Enterobacter aerogenes* | ATCC 13048 | - | - | - | - | - |
| *Enterobacter cloacae* | ATCC 13047 | - | - | - | - | - |
| *Enterococcus faecalis* | DSM 20478 | - | - | N | - | - |
| *Enterococcus faecalis* | ATCC 29212 | - | - | - | - | - |
| *Enterococcus faecium* | ATCC 29213 | - | - | - | - | - |
| *Escherichia coli* | ATCC 25922 | - | - | - | N | - |
| *Escherichia coli* | DSM 25922 | - | - | N | - | - |
| *Escherichia coli* | ATCC 35218 | - | - | - | - | - |
| *Escherichia coli* | DSM 682 | - | - | - | - | - |
| *Eubacterium plautii* | DSM 4000 | - | - | N | - | - |
| *Eubacterium tenue* | DSM 20695 | - | - | N | - | - |
| *Flavobacterium aquatile* | LMG 400g ^T^ | N | - | - | - | - |
| *Flavobacterium ferrugineum* | LMG 4021 ^T^ | N | - | - | - | - |
| *Flavobacterium odoratum* | LMG 4028 | N | - | - | - | - |
| *Flavobacteriztm odoratum* | LMG 1233 ^T^ | N | - | - | - | - |
| *Flexibacter columnaris* | LMG 13035 | N | - | - | - | - |
| *Flexitbrix dorotheae* | DSM 6795 ^T^ | N | - | - | - | - |
| *Fusobacterium nucleatum subsp. polymorphum* | DSM 20482 | - | - | N | - | - |
| *Fusobacterium nucleatum subsp. nucleatum* | ATCC 25586 ^T^ | - | P | - | - | - |
| *Haemophilus influenzae* | ATCC 3391 | - | - | - | P | - |
| *Haemophilus influenzae* | CF (5×) | - | - | - | P | - |
| *Haemophilus influenzae* | Clinical isolate | - | N | - | - | - |
| *Haemophilus parainfluenzae* | DSM 8978 | - | - | - | N | - |
| *Haliscomenobacter hydrossis* | DSM llOO^T^ | N | - | - | - | - |
| *Klebsiella pneumoniae* | DSM 3104 | - | - | - | - | - |
| *Klebsiella oxytoca* | Clinical isolate | - | - | - | - | - |
| *Lactobacillus acidophilus* | NIZO B228 | - | - | N | - | - |
| *Lactococcus lactis subsp. cremoris* | DSM 20069 | - | - | P | - | - |
| *Leuconostoc lactis* | NIZO B630 | - | - | P | - | - |
| *Porphyromonas gingivalis* | ATCC 33277 ^T^ | - | N | - | - | - |
| *Prevotella intermedia* | ATCC 25611 ^T^ | - | N | - | - | - |
| *Prevotella loescheii* | ATCC 15930 ^T^ | N | - | - | - | - |
| *Propionibacterium propionicus* | DSM 43307 | - | - | - | - | - |
| *Proteus mirabilis* | ATCC 43071 | - | - | - | - | - |
| *Proteus vulgaris* | Clinical isolate | - | - | - | - | - |
| *Pseudomonas aeruginosa* | DSM 50071 | - | - | - | N | - |
| *Pseudomonas aeruginosa* | ATCC 25853 | - | - | - | N | - |
| *Pseudomonas aeruginosa* | ATCC 10145 | - | - | - | - | - |
| *Pseudomonas aeruginosa* | CF (10×) | - | - | - | N | - |
| *Pseudomonas alcaligenes* | DSM 50342 | - | - | - | N | - |
| *Pseudomonas chlororaphis* | DSM 50083 | - | - | - | N | - |
| *Pseudomonas fluorescens* | DSM 50090 | - | - | - | N | - |
| *Pseudomonas putida* | DSM 291 | - | - | - | N | - |
| *Pseudomonas* spp. | SS266 | - | - | - | - | P |
| *Pseudomonas* spp. | SS51 | - | - | - | - | P |
| *Pseudomonas stutzeri* | DSM 5190 | - | - | - | N | - |
| *Riemerella anatipestifer* | LMG 11054 ^T^ | N | - | - | - | - |
| *Riemerella anatipestifer* | LMG 11602 | N | - | - | - | - |
| *Saprospira grandis* | DSM 2844 | N | - | - | - | - |
| *Serratia* spp. | SS301 | - | - | - | - | N |
| *Serratia* spp. | SS305 | - | - | - | - | N |
| *Sphingobacterium heparinum* | LMG 4024 ^T^ | N | - | - | - | - |
| *Sphingobacterium mizutae* | LMG 8340 ^T^ | N | - | - | - | - |
| *Sphingobacterium spiritivorum* | LMG 8347 ^T^ | N | - | - | - | - |
| *Sphingobacterium spiritivorum* | LMG 8348 | N | - | - | - | - |
| *Sporocytophaga myxococcoides* | LMG 8393 ^T^ | N | - | - | - | - |
| *Staphylococcus aureus* | ATCC 25923 | - | - | - | - | - |
| *Staphylococcus aureus* | ATCC 25423 | - | - | - | - | - |
| *Staphylococcus aureus* | ATCC 29213 | - | - | - | - | - |
| *Staphylococcus aureus* | ATCC 33862 | - | - | - | - | - |
| *Staphylococcus aureus* | DSM 346 | - | - | - | - | - |
| *Staphylococcus cohnii* | ATCC 35662 | - | - | - | - | - |
| *Staphylococcus epidermidis* | ATCC 12228 | - | - | - | - | - |
| *Staphylococcus epidermidis* | ATCC 14990 | - | - | - | - | - |
| *Staphylococcus haemolyticus* | DSM 20264 | - | - | - | - | - |
| *Staphylococcus schleiferi* | DSM 4807 | - | - | - | - | - |
| *Staphylococcus schleiferi* | DSM 6628 | - | - | - | - | - |
| *Staphylococcus sciuri* | DSM 20345 | - | - | - | - | - |
| *Stenotrophomonas maltophilia* | DSM 50170 | - | - | - | N | - |
| *Stenotrophomonas maltophilia* | CF (10×) | - | - | - | N | - |
| *Stenotrophomonas maltophilia* | ATCC 13637 | - | - | - | - | - |
| *Stenotrophomonas maltophilia* | DSM 50170 | - | - | - | - | - |
| *Streptococcus agalactiae* | DSM 2134 | - | - | - | - | - |
| *Streptococcus bovis* | MMB | - | - | P | - | - |
| *Streptococcus equi subsp.equi* | MMB | - | - | P | - | - |
| *Streptococcus equinus* | MMB | - | - | P | - | - |
| *Streptococcus intermedius* | DSM 20573 | - | - | P | - | - |
| *Streptococcus mitis* | MMB | - | - | P | - | - |
| *Streptococcus mutans* | DSM 20523 | - | - | P | - | - |
| *Streptococcus mutans* | DSM 20662 | - | - | - | - | - |
| *Streptococcus mutans* | ATCC 35668 | - | - | - | - | - |
| *Streptococcus oralis* | DSM 20627 | - | - | P | - | - |
| *Streptococcus pneumonia* | DSM 20566 | - | - | - | N | - |
| *Streptococcus pyogenes* | MMB | - | - | P | - | - |
| *Streptococcus pyogenes* | DSM 2071 | - | - | - | N | - |
| *Streptococcus pyogenes* | DSM 20565 | - | - | - | - | - |
| *Streptococcus pyogenes* | ATCC 19615 | - | - | - | - | - |
| *Streptococcus salivarius* | DSM 20560 | - | - | - | - | - |
| *Streptococcus salivarius* | MMB | - | - | P | - | - |
| *Streptococcus sanguis* | DSM 20567 | - | - | P | - | - |
| *Streptococcus sanguis* | MMB | - | - | P | - | - |
| *Streptococcus viridans* | Clinical isolate | - | N | - | - | - |
| *Tannerella forsythensis* | ATCC 43037 ^T^ | - | N | - | - | - |
| *Taxeobacter ocellatus* | GBF Txol | N | - | - | - | - |
| *Treponema denticola* | ATCC 35405 | - | N | - | - | - |
| *Treponema maltophilum* | ATCC 51939 ^T^ | - | N | - | - | - |
| *Treponema socranskii subsp. buccale* | ATCC 35534 ^T^ | - | N | - | - | - |
| *Treponema socranskii subsp. socranskii* | ATCC 35536 ^T^ | - | N | - | - | - |
| *Treponema vincentii* | ATCC 35580 | - | N | - | - | - |
| *Vagococcus fluvialis* | DSM 5731 | - | - | N | - | - |
| *Veillonella dispar* | ATCC 17748 | - | N | - | - | - |
| *Veillonella parvula* | ATCC 10790 ^T^ | - | N | - | - | - |
| *Weeksella virosa* | LMG 8350 | N | - | - | - | - |
| *Weeksella virosa* | LMG 8349 | N | - | - | - | - |
| *Weissella kandleri* | DSM 20593 | - | - | N | - | - |

Abbreviations: P = Positive hybridization signal, N = No hybridisation signal, - = not tested,

* superscript T denotes a type strain

**
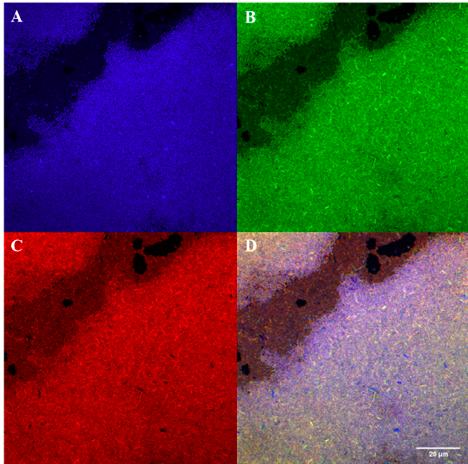
**

**Figure A.1 Fluorescence *in situ* hybridisation staining of pure *P. aeruginosa* ATCC BAA-47 pure cells from broth culture with *Pseudomonas* spp*.* probe.** (A) Nucleic material/DAPI in blue, (B) Eubacterial probe (EUB) in green, (C) *Pseudomonas* spp. specific probe (PSE277) in red, (D) composite image of (A), (B), (C). Images at 60X magnification.


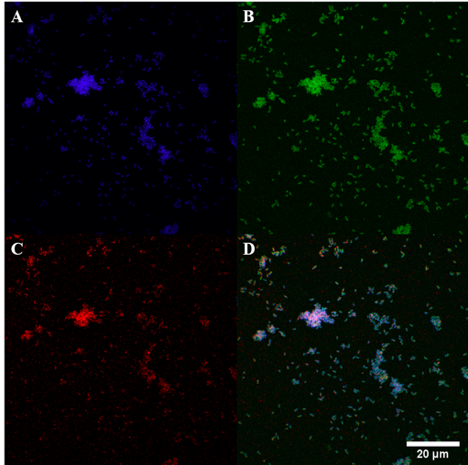


**Figure A.2 Fluorescence *in situ* hybridisation staining of *B. Fragilis* ATCC 25285 pure cells from broth culture with *Bacteroides* spp. probe.** (A) Nucleic material/DAPI in blue, (B) Eubacterial probe (EUB) in green, (C) *Bacteroides* spp. specific probe (BAC303) in red, (D) composite image of A, B & C. Images at 60X magnification.

*
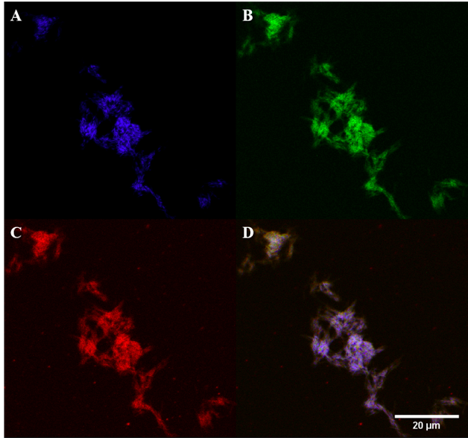
*

**Figure A.3 Fluorescence *in situ* hybridisation staining of *F. nucleatum* ATCC 25586 pure cells from broth culture with *Fusobacterium* spp. probe.** (A) Nucleic material/DAPI in blue, (B) Eubacterial probe (EUB) in green, (C) *Fusobacterium* spp. specific probe (FUSO) in red, (D) composite image of A, B & C. Images at 100X magnification.

**
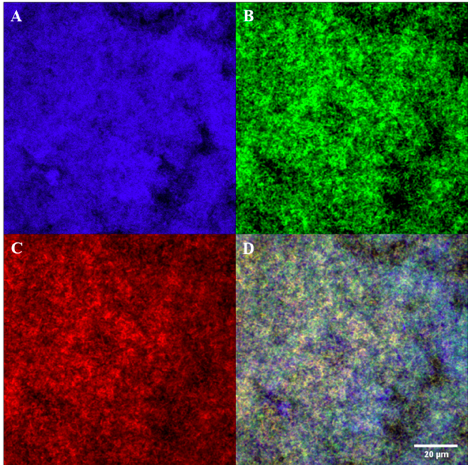
**

**Figure A.4 Fluorescence *in situ* hybridisation staining of pure *Haemophilus influenzae* ATCC 10211 type b cells from broth culture.** (A) Nucleic material/DAPI in blue, (B) Eubacterial probe (EUB) in green, (C) *H. influenzae* specific probe (Haeinf) in red, (D) composite image of A, B & C. Images at 60X magnification.

**
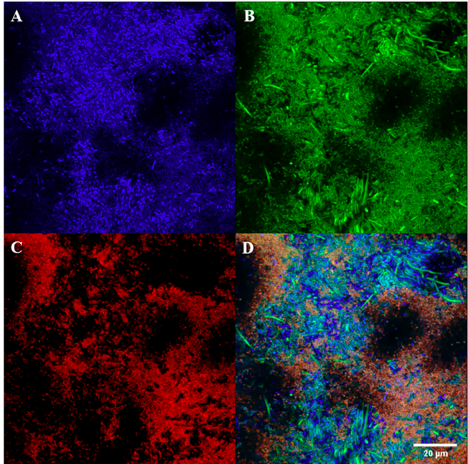
**

**Figure A.5 Fluorescence *in situ* hybridisation staining of *P. aeruginosa* ATCC BAA-47 and *E. coli* K-12 DH5 alpha, ATCC PTA-4079 mixed cells from broth culture with *Pseudomonas* spp. probe.** (A) Nucleic material/DAPI in blue, (B) Eubacterial probe (EUB) in green, (C) *Pseudomonas* spp. specific probe (PSE277) in red, (D) composite image of A, B & C. Images at 60X magnification.

**
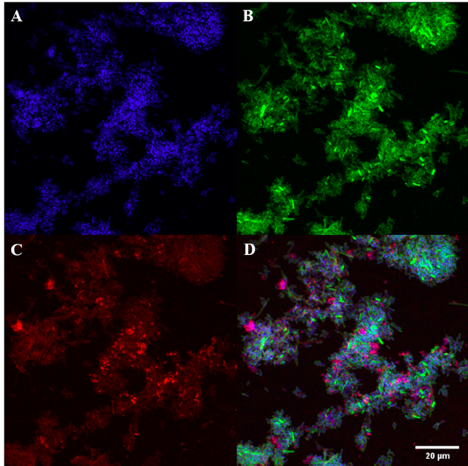
**

**Figure A.6 Fluorescence *in situ* hybridisation staining of *B. fragilis* ATCC 25285 and *E. coli* K-12 DH5 alpha, ATCC PTA-4079 mixed cells from broth culture with *Bacteroides* spp. probe.** (A) Nucleic material/DAPI in blue, (B) Eubacterial probe (EUB) in green, (C) *Bacteroides* spp. specific probe (BAC303) in red, (D) composite image of A, B & C. Images at 60X magnification.


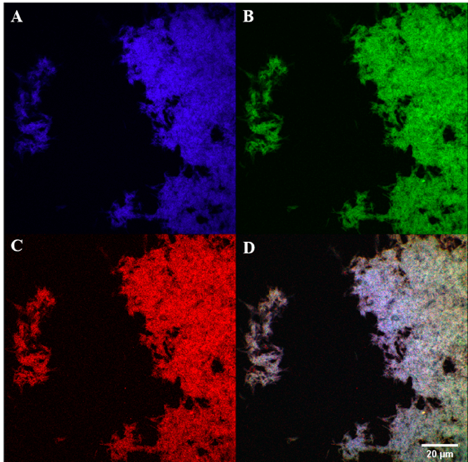


**Figure A.7 Fluorescence *in situ* hybridisation staining of *F. nucleatum* ATCC 25586 and *E. coli* K-12 DH5 alpha, ATCC PTA-4079 mixed cells from broth culture with *Fusobacterium* spp. probe.** (A) Nucleic material/DAPI in blue, (B) Eubacterial probe (EUB) in green, (C) *Fusobacterium* spp. specific probe (FUSO) in red, (D) composite image of A, B & C. Images at 60X magnification.

**
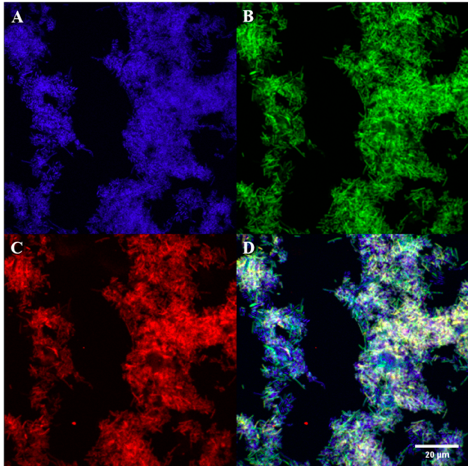
**

**Figure A.8 Fluorescence *in situ* hybridisation staining of mixed *Haemophilus influenzae* ATCC 10211 type b and *E. coli* K-12 DH5 alpha, ATCC PTA-4079 mixed cells from broth culture with *Haemophilus influenzae* species-specific probe.** (A) Nucleic material/DAPI in blue, (B) Eubacterial probe (EUB) in green, (C) *H. influenzae* species-specific probe (Haeinf) in red, (D) composite image of A, B & C. Images at 60X magnification.

**
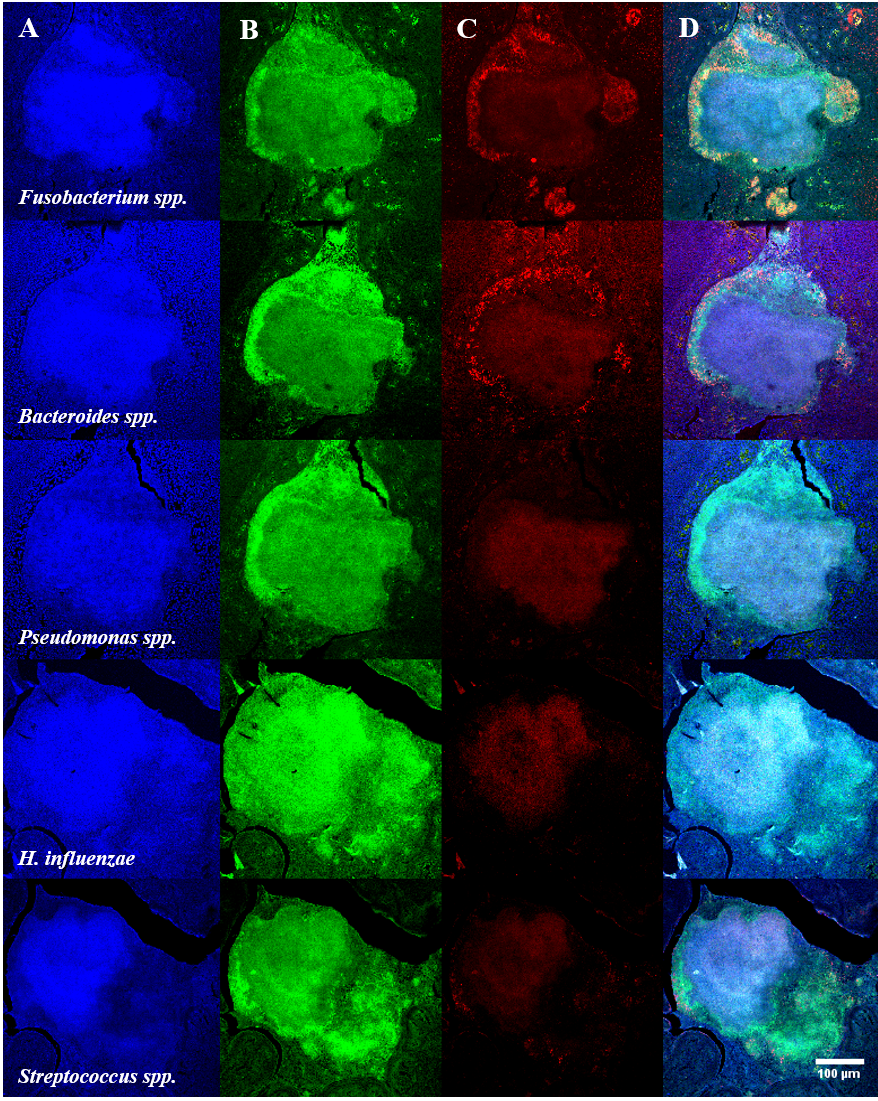
**

**Figure A.9 Fluorescence *in situ* hybridisation staining of *Fusobacterium* spp.*, Bacteroides* spp.*, Pseudomonas* spp.*, H. influenzae* and *Streptococcus* spp. specific probes in the same bacterial microcolony in a child with RT.**

Rows: *Fusobacterium* spp., *Bacteroides* spp., *Pseudomonas* spp., *H. influenzae* and *Streptococcus* spp. specific probes. Columns: (A) Nucleic material/DAPI in blue, (B) Eubacterial probe (EUB) in green, (C) Specific bacterial taxa probe in red, (D) composite image of (A), (B), (C). Images at 10X magnification.
